# Real-world outcomes on platinum-containing chemotherapy for *EGFR*-mutated advanced nonsquamous NSCLC with prior exposure to EGFR tyrosine kinase inhibitors

**DOI:** 10.3389/fonc.2024.1285280

**Published:** 2024-04-18

**Authors:** Balazs Halmos, Pragya Rai, Jae Min, Xiaohan Hu, Diana Chirovsky, Mark Shamoun, Bin Zhao

**Affiliations:** ^1^ Department of Oncology, Montefiore Medical Center/Albert Einstein College of Medicine, Bronx, NY, United States; ^2^ Center for Observational and Real-World Evidence, Merck & Co., Inc., Rahway, NJ, United States; ^3^ Clinical Research, Merck & Co., Inc., Rahway, NJ, United States

**Keywords:** advanced non-small cell lung cancer, sensitizing *EGFR* mutation, overall survival, platinum-containing chemotherapy, subsequent therapy, tyrosine kinase inhibitor

## Abstract

**Background:**

Front-line therapy with an EGFR tyrosine kinase inhibitor (TKI) is the standard of care for treating patients with advanced nonsquamous NSCLC with the common sensitizing *EGFR* exon 19 deletion and exon 21 L858R point mutations. However, EGFR TKI resistance inevitably develops. The optimal subsequent therapy remains to be identified, although platinum-containing chemotherapy regimens are often administered. Our objectives were to describe baseline characteristics, survival, and subsequent treatment patterns for patients with advanced nonsquamous NSCLC with *EGFR* exon 19 deletion or L858R mutation who received a platinum-based combination regimen after front-line EGFR TKI therapy.

**Methods:**

This retrospective study used a nationwide electronic health record-derived deidentified database to select adult patients with advanced nonsquamous NSCLC, evidence of *EGFR* exon 19 deletion or L858R mutation, and ECOG performance status of 0-2 who initiated platinum-containing chemotherapy, with or without concomitant immunotherapy, from 1-January-2011 to 30-June-2020 following receipt of any EGFR TKI as first-line therapy or, alternatively, a first- or second-generation EGFR TKI (erlotinib, afatinib, gefitinib, dacomitinib) as first-line therapy followed by the third-generation EGFR TKI osimertinib as second-line therapy. Data cut-off was 30-June-2022. The Kaplan-Meier method was used to estimate overall survival (OS) after initiation of pemetrexed-platinum (n=119) or any platinum-based combination regimen (platinum cohort; n=311).

**Results:**

The two cohorts included two-thirds women (65%-66%) and 57%-58% nonsmokers; median ages were 66 and 65 years in pemetrexed-platinum and platinum cohorts, respectively. Median OS was 10.3 months (95% CI, 8.1-13.9) from pemetrexed-platinum initiation and 12.4 months (95% CI, 10.2-15.2) from platinum initiation; 12-month survival rates were 48% and 51%, respectively; 260 patients (84%) had died by the end of the study.

**Conclusion:**

The suboptimal survival outcomes recorded in this study demonstrate the unmet need to identify more effective subsequent treatment regimens for patients with *EGFR*-mutated advanced nonsquamous NSCLC after EGFR TKI resistance develops.

## 1 Introduction

Knowledge of the molecular biology of non-small cell lung cancer (NSCLC) has rapidly expanded in recent years, together with the development of therapies targeting specific oncogenic drivers (1). Mutations in the epidermal growth factor receptor (*EGFR*) gene are common in lung adenocarcinoma, with an estimated prevalence in lung adenocarcinoma of 23% (range, 3–42%) in the United States (US) and greater frequency in Asian than non-Asian patients, in women than men, and in never-smokers (*vs*. smokers) (2, 3). The most common sensitizing *EGFR* mutations are exon 19 deletions (ex19del) and exon 21 L858R point mutations (3–6).

Testing for *EGFR* mutations in the advanced NSCLC setting came into standard practice as early as 2011, when clinical trials were examining the place in therapy of the first-generation EGFR tyrosine kinase inhibitors (TKIs) gefitinib and erlotinib (7). These agents, as well as the second-generation EGFR TKIs afatinib and dacomitinib and the third-generation EGFR TKI osimertinib have demonstrated superiority over chemotherapy for treating *EGFR*-mutated NSCLC as front-line therapy (6, 8). Osimertinib was originally approved for treating the *EGFR* T790M mutation in exon 20, the most common mutation conferring resistance to first- and second-generation EGFR TKIs (4, 6). Front-line therapy with osimertinib is current standard of care for patients with stage IV NSCLC with *EGFR* ex19del or L858R mutation and Eastern Cooperative Oncology Group performance status (ECOG PS) of 0–2 based on the FLAURA trial results demonstrating improved outcomes over first-generation agents (9). However, drug resistance typically develops also with osimertinib. Diverse resistance mechanisms to the EGFR TKIs include on-target alterations, bypass signaling pathway activation, and histological transformation (6, 10).

Identifying the optimal subsequent therapy for patients who experience disease progression after EGFR TKI therapy remains an active area of study (11–14). Clinical trial results suggest no benefit of adding immunotherapy to platinum-based therapies for treating TKI-resistant metastatic NSCLC (15–18), with the exception of a small subgroup analysis of patients with *EGFR*-mutated NSCLC and prior TKI exposure who received atezolizumab added to a bevacizumab-carboplatin-paclitaxel regimen (19), a regimen that nonetheless did not garner US regulatory approval. Moreover, recently reported interim analyses of the ORIENT-31 trial suggest benefits in progression-free survival for patients receiving sintilimab plus chemotherapy, with or without bevacizumab biosimilar IBI305, as compared with chemotherapy alone (20, 21). Current clinical guidelines list multiple regimens, most of them platinum-based, to consider after EGFR TKI resistance develops (9, 22, 23).

Data from real-world oncology practice can provide information to supplement clinical trial data (24, 25). Recent observational studies have examined first-line EGFR TKI treatment patterns and outcomes for patients with *EGFR*-mutated advanced NSCLC (26–28); however, information remains limited regarding the outcomes of platinum-based regimens administered after disease progression on EGFR TKI therapy in real-world settings (29). The aims of this study were to describe baseline characteristics, survival, and subsequent treatment patterns for patients with advanced nonsquamous NSCLC with *EGFR* ex19del or L858R mutation who received pemetrexed plus platinum or other platinum-based combination regimen as the next line of therapy after front-line EGFR TKI therapy at US oncology practices.

## 2 Methods

### 2.1 Data source and patients

The nationwide Flatiron Health database contains deidentified, electronic health record-derived patient-level data from oncology practices throughout the US, including approximately 280 cancer clinics (~800 sites of care) at the time of this study. The longitudinal, structured and unstructured data are curated via technology-enabled abstraction and include patient characteristics and lines of systemic anticancer therapy defined by oncologist-defined, rules-based methods, as previously described (30–32).

We studied patients in the Flatiron Health advanced NSCLC database, which includes patients with at least two recorded clinic visits and a pathologically confirmed diagnosis of advanced NSCLC (unresectable stage IIIB/IIIC, or stage IV) on or after 1 January 2011. Eligible patients were ≥18 years old with nonsquamous NSCLC and evidence of *EGFR* ex19del or L858R mutation. We selected those who initiated platinum-containing chemotherapy from 1 January 2011 to 30 June 2020 after having received EGFR TKI therapy.

Eligible patients had received any EGFR TKI as first-line therapy or, alternatively, a first- or second-generation EGFR TKI (erlotinib, afatinib, gefitinib, dacomitinib) as first-line therapy followed by osimertinib in second line. From these patients, we then selected those who received combination pemetrexed plus platinum (pemetrexed-platinum cohort) as subsequent therapy. Because patients with progression after an EGFR TKI can also receive other platinum combination therapies, we selected a second cohort that included all patients who received any platinum-based combination regimen, with or without concomitant immunotherapy (platinum cohort). Thus, the platinum cohort also included patients who received pemetrexed-platinum, and the two cohorts were not mutually exclusive. (Platinum was defined as carboplatin or cisplatin.)

Patients enrolled in a clinical trial or with ECOG PS of ≥3 were excluded. In addition, we excluded those who had no clinic visit ≤90 days after the advanced NSCLC diagnosis, as a potential indication of insufficient follow-up. The data cutoff date was 30 June 2022, allowing for a minimum follow-up period of 2 years from the time of initiating platinum-containing chemotherapy.

Institutional Review Board approval of the study protocol was obtained before conducting the study and included a waiver of informed consent for working with deidentified data. The deidentified data were subject to obligations to prevent reidentification during the analyses to protect patient confidentiality.

### 2.2 Outcomes and analyses

We summarized patient demographics and clinical characteristics at initiation of platinum-containing chemotherapy (defined as the “index date”) for the pemetrexed-platinum cohort and the platinum cohort. The baseline ECOG PS was identified as that recorded closest to the index date and within 30 days before to 30 days after the index date. In addition, we summarized the platinum-containing regimen types and the regimens administered in the next line of therapy (namely, third line or fourth line, depending whether osimertinib was administered in second line).

The Kaplan-Meier method was used to estimate median overall survival (OS) with 95% confidence intervals (CIs) and survival rates at 12 and 24 months; patients who were alive at data cutoff were censored. Dates of death were determined using a validated real-world mortality endpoint (33–35). We also conducted a sensitivity analysis to estimate OS for patients with ECOG PS of 0 or 1.

All patients who met eligibility criteria were included in descriptive and Kaplan-Meier survival analyses. No formal sample size or power calculations were conducted; and SAS software, version 9.4 (SAS Institute, Cary, NC) was used for the analyses.

## 3 Results

### 3.1 Patients and therapy

**Figure 1:**
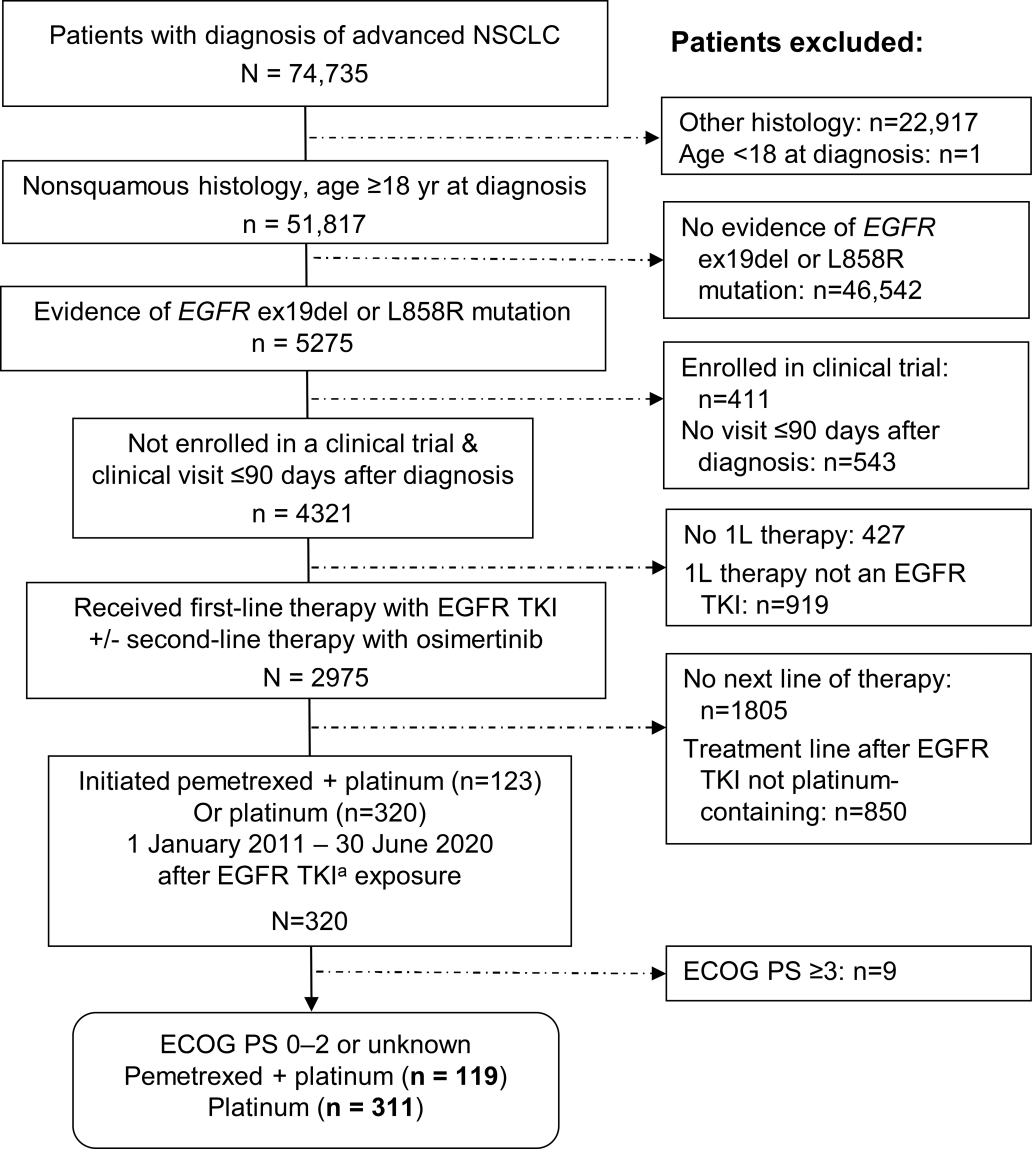
Selection of eligible patients from the deidentified database. ^a^EGFR TKI exposure included any EGFR TKI as first-line therapy or first-/second-generation EGFR TKI in first line and third-generation EGFR TKI (osimertinib) in second line. 1L, 2L, 3L, first-, second-, third-line therapy; ECOG PS, Eastern Cooperative Oncology Group performance status; EGFR ex19del, EGFR exon 19 deletion; TKI, tyrosine kinase inhibitor.

The deidentified database included 2975 patients with *EGFR*-mutated nonsquamous NSCLC who received a first-line EGFR TKI with or without second-line osimertinib (**Figure 1**). Of these 2975 patients, 320 (11%) had a record of then initiating a platinum-containing regimen from 2011 to mid-2020. After excluding 9 patients (3%) with ECOG PS of 3 or 4, we studied 119 patients in the pemetrexed-platinum cohort and 311 patients in the platinum cohort (**Figure 1**).

Baseline demographic characteristics of patients in the two cohorts were similar. Median ages were 66 and 65 years, including 20% and 19% aged ≥75 years and 65% and 66% women in the pemetrexed-platinum and platinum cohorts, respectively (**Table 1**). Approximately three-quarters of patients with known race were White, 6% to 8% were Black, while 18% in the pemetrexed-platinum and 15% in the platinum cohort were Asian. The majority of patients were treated at community oncology practices (78% and 82%, respectively).

**Table 1:** Characteristics at the index date of patients with *EGFR*-mutated nonsquamous advanced NSCLC previously treated with an EGFR TKI^a^.

Characteristic	Pemetrexed-Platinum (N = 119)	Platinum^b^ (N = 311)
Age, median (range), years	66 (31–81)	65 (31–81)
Age group		
<75 years	95 (79.8)	252 (81.0)
≥75 years	24 (20.2)	59 (19.0)
Sex, female	77 (64.7)	206 (66.2)
Race^c^		
White	70 (63.1)	189 (65.6)
Asian	20 (18.0)	44 (15.3)
Black or African American	7 (6.3)	23 (8.0)
Other	14 (12.6)	32 (11.1)
Unknown	8	23
Practice type		
Community	93 (78.2)	254 (81.7)
Academic	23 (19.3)	49 (15.8)
Both community and academic	3 (2.5)	8 (2.6)
Smoking status^c^		
Positive history of smoking	50 (42.7)	129 (41.7)
No history of smoking	67 (57.3)	180 (58.3)
Unknown	2	2
ECOG performance status at the index date^c^		
0–1	61 (76.2)	177 (83.9)
2	19 (23.8)	34 (16.1)
Unknown	39	100
Charlson comorbidity index		
Mean (SD)	6.0 (3.4)	6.1 (3.2)
Median (range)	8 (0–16)	8 (0–16)
Record of brain metastasis^d^	26 (21.8)	57 (18.3)
Advanced stage at initial diagnosis^c,e^	105 (89.7)	278 (90.6)
*EGFR* mutation type		
* EGFR* exon 19 deletion	70 (58.8)	191 (61.4)
* EGFR* exon 21 L858R mutation	49 (41.2)	119 (38.3)
Both *EGFR* ex19del & L858R mutation	0	1 (0.3)
PD-L1 expression^c^		
≥50%	6 (14.0)	29 (21.0)
1–49%	12 (27.9)	53 (38.4)
<1%	25 (58.1)	56 (40.6)
Unknown	76	173
Index year^a^		
2011–2015	45 (37.8)	103 (33.1)
2016–2020	74 (62.2)	208 (66.9)
Line of osimertinib		
None	78 (65.5)	190 (61.1)
1L	20 (16.8)	55 (17.7)
2L	21 (17.6)	66 (21.2)
Index platinum-containing line of therapy^a^		
2L	98 (82.4)	245 (78.8)
3L (after 2L osimertinib)	21 (17.6)	66 (21.2)

Data are n (%) unless otherwise noted. Percentages may not add up to 100 because of rounding.

a The index date was defined as the date of initiating platinum-containing chemotherapy.

b Platinum-containing regimen with or without concomitant immunotherapy as index therapy.

c Percentages for race, smoking status, ECOG performance status, advanced stage at initial diagnosis, and PD-L1 expression represent the percentages of patients with available data. ECOG performance status was determined within 30 days before or after the index date.

d Information was not available regarding whether brain metastases were active or previously treated.

e Advanced stage at initial diagnosis included stages IIIB, IIIC, and IV. (Six patients had no recorded stage at initial diagnosis.)

1L, 2L, 3L, first-, second-, third-line of therapy. ECOG, Eastern Cooperative Oncology Group; PD-L1, programmed death-ligand 1; SD, standard deviation.

Most patients (90% and 91%, respectively) received an initial diagnosis of NSCLC at an advanced stage, while fewer than half of patients had a history of smoking (**Table 1**). In the pemetrexed-platinum and platinum cohorts, 22% and 18%, respectively, had a record of brain metastasis.

The *EGFR* mutations were identified most commonly via tissue samples (data not shown). One patient in the platinum cohort had a record of both *EGFR* ex19del and L858R mutation; all other patients had tumors with either *EGFR* ex19del or L858R mutation (**Table 1**).

The distribution of PD-L1 expression is summarized in **Table 1** for the half or fewer patients in each cohort who had recorded results: namely, 36% and 44% of patients in the pemetrexed-platinum and platinum cohorts, respectively. The platinum-containing therapies are detailed in **Supplementary Table 1**. Overall, 87 of 311 patients (28%) received concomitant immunotherapy.

### 3.2 Outcomes

The median follow-up from the index date to data cutoff was 65 and 59 months in pemetrexed-platinum and platinum cohorts, respectively, while median patient follow-up from the index date to the earliest of death or data cutoff was 9 and 11 months, respectively (**Table 2**).

**Table 2:** Overall survival after initiation of platinum-containing regimen.

Variable	Pemetrexed-Platinum (N = 119)	Platinum^a^ (N = 311)
Theoretical follow-up, median (range), mo^b^	65.3 (24.8–133.3)	58.8 (24.8–133.5)
Patient follow-up, median (range), mo^b^	9.4 (0.1–90.6)	11.2 (<0.1–107.6)
Overall survival (OS), events, n (%)	103 (86.6)	260 (83.6)
Median OS (95% CI), months	10.3 (8.1–13.9)	12.4 (10.2–15.2)
OS rate, % (95% CI)		
At 12 months	47.5 (38.2–56.3)	51.2 (45.4–56.7)
At 24 months	23.3 (15.8–31.7)	27.5 (22.4–32.8)

Patients with ECOG PS 0 or 1	N=61	N=177
Overall survival (OS), events, n (%)	48 (78.7)	142 (80.2)
Median OS (95% CI), months	13.6 (9.2–23.1)	14.5 (11.1–17.9)
OS rate, % (95% CI)		
At 12 months	55.7 (42.0–67.3)	55.9 (48.2–63.0)
At 24 months	31.2 (19.2–43.9)	31.6 (24.5–38.9)

ECOG PS, Eastern Cooperative Oncology Group performance status.

a Platinum-containing regimen with or without concomitant immunotherapy as index therapy.

b Theoretical follow-up was defined as the time from the index date to data cut-off, and patient follow-up was defined as the time from the index date to the earliest of death, last visit date, or data cutoff.

**Figure 2:**
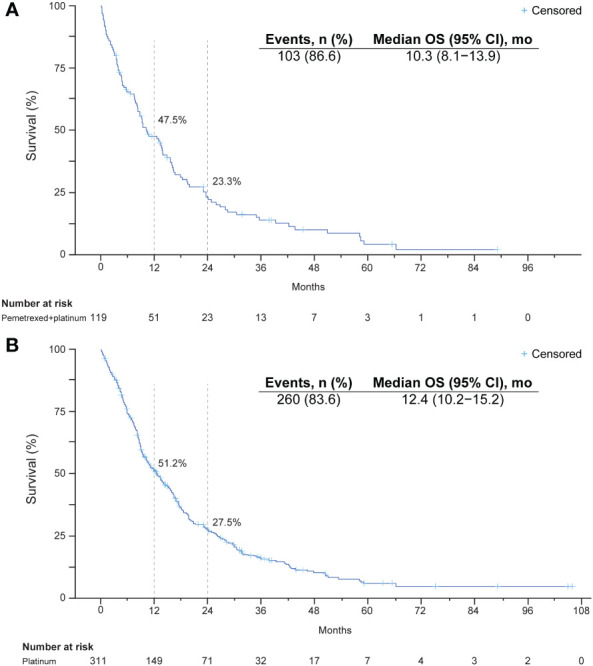
Kaplan-Meier plots of overall survival after initiation of a platinum-containing regimen: **(A)** platinum-pemetrexed combination regimen and **(B)** platinum-based regimen with or without immunotherapy.

Median OS from initiation of pemetrexed-platinum was 10.3 months (95% CI, 8.1–13.9), and the survival rates at 12 and 24 months were 48% and 23%, respectively (**Table 2**, **Figure 2**). In the platinum cohort, median OS was 12.4 months (95% CI, 10.2–15.2), and the survival rates at 12 and 24 months were 51% and 28%, respectively.

Among the patients with ECOG PS of 0 or 1, median survival was 13.6 months in the pemetrexed-platinum cohort and 14.5 months in the platinum cohort (**Table 2**).

### 3.3 Systemic treatment regimens received after platinum-containing regimens

Seventy-one of 119 patients (60%) in the pemetrexed-platinum cohort and 201 of 311 patients (65%) in the platinum cohort received another systemic therapy after the platinum-containing regimen. These included PD-(L)1 inhibitor-, EGFR TKI-, and taxane-based regimens (**Table 3**).

**Table 3:** Systemic treatment regimens received after the platinum-containing regimen.

Treatment-related variable	Pemetrexed-Platinum (N = 119)	Platinum^a^ (N = 311)
2L platinum-containing LOT, n	98	245
3L after 2L platinum-containing LOT^b^	61 (62.2)	164 (66.9)
PD-(L)1 inhibitor monotherapy	16 (26.2)	35 (21.3)
PD-(L)1 inhibitor-chemotherapy	5 (8.2)	14 (8.5)
Taxane monotherapy	9 (14.8)	15 (9.1)
Taxane-containing combination regimen	7 (11.5)	14 (8.5)
EGFR TKI	18 (29.5)	59 (36.0)
Osimertinib-containing regimen^c^	10	30
Afatinib/erlotinib/gefitinib	8	29
Other^d^	6 (9.8)	27 (16.5)
3L platinum-containing LOT, n	21	66
4L after 3L platinum-containing LOT^b^	10 (47.6)	37 (56.1)
PD-(L)1 inhibitor monotherapy	6 (60.0)	10 (27.0)
PD-(L)1 inhibitor-chemotherapy	0	3 (8.1)
PD-1+PD-L1 inhibitor-combination	1 (10.0)	1 (2.7)
Taxane monotherapy	1 (10.0)	3 (8.1)
Taxane-containing combination regimen	1 (10.0)	8 (21.6)
EGFR TKI	1 (10.0)	7 (18.9)
Osimertinib-containing regimen^c^	1	5
Afatinib/erlotinib/gefitinib/dacomitinib	0	2
Other^d^	0	5 (13.5)

Data are n (%) unless otherwise noted. Percentages may not total 100 because of rounding.

a Platinum-containing regimen with or without immunotherapy as index therapy.

b Drug regimens in 3L and 4L are shown as percentages of the relevant treatment line. For each line of therapy, mutually exclusive regimen classes were assigned in hierarchical order as follows: PD-(L)1 inhibitor-based therapy > taxane-containing regimen or EGFR TKI > other therapy.

c Osimertinib-containing regimens included osimertinib monotherapy and combination therapy.

d The ‘Other’ category included 15 different regimens administered as a monotherapy (eg, pemetrexed or gemcitabine) or combination therapy (eg, carboplatin-pemetrexed).

2L, 3L, 4L, second-, third-, fourth-line of therapy (LOT); PD-(L)1, programmed death-(ligand)1.

## 4 Discussion

This retrospective study followed patients treated in the real-world setting of US oncology practices for advanced nonsquamous NSCLC with sensitizing *EGFR* mutations (ex19del/L858R) and who initiated platinum-containing chemotherapy from 2011 to mid-2020 after having received one or two lines of EGFR TKI therapy, with follow-up to mid-2022. Median OS after initiating pemetrexed-platinum was 10.3 months and after platinum, 12.4 months; 12-month survival rates were 48% and 51%, respectively. Outcomes were somewhat better for patients with good performance status, namely, median OS of 13.6 months and 14.5 months after pemetrexed-platinum and platinum initiation, respectively, with 12-month survival rates of 56% in both cohorts. Subsequent third- and fourth-line therapies were varied and included PD-(L)1 inhibitor-based, taxane-based, and EGFR TKI-based regimens.

Real-world outcomes were also examined in a small retrospective study of 135 patients with *EGFR*-mutated advanced NSCLC who received subsequent therapy in 2015 to 2021 after EGFR TKIs. Treated at two tertiary cancer centers in the Netherlands, these patients received a variety of chemotherapy regimens; and median OS was 15.3 months (95% CI, 11.6–18.9), slightly longer than in the present study, with no significant difference detected among regimens, the most common of which was pemetrexed-platinum (36).

In a recent US real-world study, Nieva et al. used the CancerLinQ database to examine treatment patterns and survival of patients with *EGFR*-mutated advanced NSCLC diagnosed in 2011–2018 who received first-line therapy with a first- or second-generation EGFR TKI (29). Median OS from the start of second-line treatment was longer for the 186 patients who received osimertinib in second-line (28.9 months) than for the 353 who received other therapies (13.0 months), the minority of whom received platinum-based chemotherapy (as in our study). Of note, in their study, 28% of patients died before initiating second-line treatment, and only 52% of patients continued to second-line. Similar to our study, NSCLC was diagnosed at an advanced stage for the majority of patients (29). Other recent US real-world studies of patients with *EGFR-*mutated NSCLC have described biomarker testing patterns and/or treatment patterns for first-line therapy and disease progression after first line (26–28, 37–41).

In recently published clinical trials of similar patient populations who experienced disease progression after EGFR TKI therapy (11–13), the median OS was somewhat longer in the pemetrexed-platinum arms (17.9, 18.7, and 19.5 months) relative to our findings for patients with ECOG PS of 0 or 1, albeit with the caveat that fewer than 50 patients were included in the treatment arms of two of the trials (12, 13). However, a better outcome in clinical trials (relative to our real-world findings) is not an unexpected finding, because of more frequent front-line osimertinib and the fact that trial enrollment criteria tend to select for patients without comorbidity or concomitant therapies, different from an unselected real-world patient population (24).

The patient population included in the present study was representative of a population with *EGFR*-mutated advanced nonsquamous NSCLC, namely, including more women (66%) than men, more Asian patients (15%) than in other US real-world studies not restricted to *EGFR-*mutated NSCLC, and more nonsmokers (57%) than smokers (3, 6, 42). We studied over 300 patients and treatment outcomes over a decade during which EGFR TKIs became the standard of care as first-line therapy for *EGFR*-mutated NSCLC. Designed with a minimum of 2 years of potential follow-up (until 30 June 2022), our study used a well-maintained database that is frequently used for oncology research (30–32).

We note several study limitations, however. Our findings may have limited generalizability to academic compared with community centers and to centers outside the Flatiron Health network (43, 44). A small percentage of Black patients (≤8%), fewer than the US population percentage of 14%, were included in the database, and ECOG PS data were missing for approximately one-third of patients. Testing for PD-L1 expression was not introduced until 2015; therefore, incomplete availability of PD-L1 expression status was not unexpected. Consistent with clinical guidelines, few patients received concomitant immunotherapy. However, the numbers (87/311) were insufficient for outcomes analysis in these patients. Finally, more recent real-world data would likely reveal different treatment patterns as osimertinib is now standard of care in front line, and further observational studies of patients treated in real-world settings are needed.

Research continues to identify the optimal systemic regimens for patients with TKI-resistant *EGFR*-mutated NSCLC. While the role of immunotherapy remains uncertain (14), several ongoing trials are investigating novel therapies, such as antibody-drug conjugates, to improve outcomes for these patients (45).

## 5 Conclusions

Survival outcomes in this real-world study of patients with advanced nonsquamous NSCLC who experienced disease progression after EGFR TKI therapy appeared shorter than, but within the range of, historical clinical trials. The wide variability in platinum-containing regimens and in regimens administered as the next line of therapy is indicative of the lack of treatment regimens with proven efficacy for this population. The suboptimal survival outcomes recorded in this study demonstrate the unmet need to identify more effective subsequent treatment regimens for patients with *EGFR*-mutated advanced nonsquamous NSCLC after EGFR TKI resistance develops.

## Data availability statement

The data that support the findings of this study have been originated by Flatiron Health, Inc. Requests for data sharing by license or by permission for the specific purpose of replicating results in this article can be submitted to publicationsdataaccess@flatiron.com.

## Ethics statement

Institutional Review Board approval of the study protocol was obtained from WCG Institutional Review Board before conducting the study and included a waiver of informed consent for working with deidentified data. The deidentified data were subject to obligations to prevent reidentification during the analyses to protect patient confidentiality. 

## Author contributions

BH: Writing – review & editing, Visualization, Validation. PR: Writing – review & editing, Writing – original draft, Visualization, Validation, Supervision, Project administration, Methodology, Conceptualization. JM: Writing – review & editing, Validation, Investigation, Formal analysis, Data curation. XH: Writing – review & editing, Visualization, Validation, Methodology, Investigation, Conceptualization. DC: Writing – review & editing, Visualization, Validation, Methodology, Conceptualization. MS: Writing – review & editing, Visualization, Validation. BZ: Writing – review & editing, Visualization, Validation.
