# Supplementary material for: Real-world outcomes on platinum-containing chemotherapy for EGFR-mutated advanced nonsquamous NSCLC with prior exposure to EGFR tyrosine kinase inhibitors
Source: Front Oncol. 2024 Apr 18;14:1285280. doi: 10.3389/fonc.2024.1285280 (PMC11063374; doi:10.3389/fonc.2024.1285280)
Supplement: Supplementary file 1 [file Table_1.docx]

**Supplementary TABLE 1 |** Platinum-containing regimens administered in second or third line

|  | **N=311** |
| --- | --- |
| **Second-line platinum-containing regimen, n** | **245** |
| Platinum-pemetrexed ± bevacizumab | 148 (60.4) |
| Carboplatin, pemetrexed | 95 |
| Bevacizumab, carboplatin, pemetrexed | 50 |
| Cisplatin, pemetrexed | 3 |
| PD(L)1 inhibitor + platinum-based regimen | 56 (22.9) |
| Carboplatin, pembrolizumab, pemetrexed | 43 |
| Atezolizumab, bevacizumab, carboplatin, paclitaxel | 7 |
| Atezolizumab, carboplatin, paclitaxel protein-bound | 2 |
| Atezolizumab, bevacizumab-bvzr, carboplatin, paclitaxel | 1 |
| Atezolizumab, carboplatin, etoposide | 1 |
| Atezolizumab, carboplatin, paclitaxel | 1 |
| Carboplatin, pembrolizumab | 1 |
| Platinum-taxane ± bevacizumab | 28 (11.4) |
| Bevacizumab, carboplatin, paclitaxel | 12 |
| Carboplatin, paclitaxel | 9 |
| Carboplatin, paclitaxel protein-bound | 4 |
| Bevacizumab, carboplatin, paclitaxel protein-bound | 3 |
| Other platinum-containing regimen | 13 (5.3) |
| Carboplatin, etoposide | 3^a^ |
| Carboplatin, gemcitabine | 3 |
| Carboplatin | 2 |
| Bevacizumab, carboplatin, docetaxel | 1 |
| Bevacizumab, cisplatin, gemcitabine | 1 |
| Carboplatin, docetaxel | 1 |
| Carboplatin, gemcitabine, pemetrexed | 1 |
| Cisplatin | 1 |
| **Third-line platinum-containing regimens, n** | **66** |
| Platinum-pemetrexed ± bevacizumab | 30 (45.5) |
| Carboplatin, pemetrexed | 21 |
| Bevacizumab, carboplatin, pemetrexed | 8 |
| Bevacizumab-bvzr, carboplatin, pemetrexed | 1 |
| PD(L)1 inhibitor + platinum-based regimen | 31 (47.0) |
| Carboplatin, pembrolizumab, pemetrexed | 27 |
| Atezolizumab, bevacizumab, carboplatin, paclitaxel | 1 |
| Atezolizumab, bevacizumab, carboplatin, pemetrexed | 1 |
| Atezolizumab, bevacizumab-awwb, carboplatin, paclitaxel | 1 |
| Carboplatin, etoposide, nivolumab | 1 |
| Platinum-taxane ± bevacizumab | 3 (4.5) |
| Carboplatin, paclitaxel | 2 |
| Bevacizumab, carboplatin, paclitaxel | 1 |
| Other platinum-containing regimen | 2 (3.0) |
| Carboplatin | 1 |
| Carboplatin, etoposide^a^ | 1^a^ |

^a^Carboplatin-etoposide is typically given for small cell transformation; however, this information was not available from the database.

The percentages for each regimen category represent the percentages for that line of platinum-containing therapy.
